# The Relationship Between Career Social Support and Employability of College Students: A Moderated Mediation Model

**DOI:** 10.3389/fpsyg.2020.00028

**Published:** 2020-01-28

**Authors:** Tiansheng Xia, Honglei Gu, Yamei Huang, Qian Zhu, Yufang Cheng

**Affiliations:** ^1^School of Art and Design, Guangdong University of Technology, Guangzhou, China; ^2^Cognition and Human Behavior Key Laboratory of Hunan Province, Department of Psychology, Hunan Normal University, Changsha, China; ^3^School of Education, Zhengzhou University, Zhengzhou, China; ^4^School of Administrative Law, Northwest University of Political Science and Law, Xi'an, China; ^5^Continuing Education College, Hunan Normal University, Changsha, China

**Keywords:** career social support, career adaptation, proactive personality, moderated mediation, employability

## Abstract

This study examines the underlying mechanism that connects career social support with employability through a survey of 392 Chinese college students. The results showed that career social support had a positive effect on career adaptation and employability of college students, and career adaptation mediated the association between career social support and employability. Furthermore, proactive personality was found to play a moderating role in linking career adaptation and employability. More specifically, higher levels of a proactive personality strengthen the enhancing effect of career adaptation on the employability of college students. Therefore, there was a moderated mediation effect between career social support and employability of college students.

## Introduction

The employment of college students is now a persistent hot topic in China. Since the expanded enrollment in 1999, the number of Chinese college graduates has increased sharply during recent years. According to a survey conducted by China’s Ministry of Education (Mycos Research Institute, 2019), the number of college graduates in China reached over 8.3 million in 2019, which was a historical high. The employment rate of college graduates was 91.5% in 2018, and it was lower than that in 2014 (92.6%), indicating that the employment rate in China was on a gentle, but steady, downward slide. Among the factors influencing successful employment, employers usually regard the candidates’ employability as a fundamental determinant of recruitment (Guilbert et al., 2016). Mcardle et al. (2007) propose that the lack of employability is the primary cause of employment difficulty for college students. Therefore, it is worthwhile to identify the influential factors that underpin undergraduates’ employability, which will be beneficial to improve the employability of college students and alleviate the social problems of unemployment.

Employability is conceptualized as a set of human capital, social capital, personal characteristics, and personal behaviors that makes graduates more likely to gain employment (Clarke, 2018). From the inputs point, employability associates with factors, such as competency, which increase the likelihood of getting and maintaining a job (De Cuyper et al., 2012). Yu et al. (2014) proposed that employability consisted of eight factors: interpersonal relationships, team cooperation, learning ability, resolving problems, social support, network difference, an optimistic and open personality, and career identification. The interpersonal relationships, team cooperation, learning ability, and resolving problems factors were competencies related to coping with interpersonal relationship and problem solving. Social support and network difference reflected the social capital, and an optimistic and open personality reflected positive personality characteristics.

Fugate et al. (2004) define employability as a person-centered, psycho-social construct, and propose that people with well-developed social and human capital often utilize formal (e.g., parents) and informal support systems (e.g., friend of a friend) to devote to the job search behaviors. According to Resource Conservation Theory (Hobfoll et al., 2003), individuals will always strive to maintain and utilize resources that contribute to their successful employment, and the more social psychological resources (e.g., support from parents and friends, and power) can result in a greater chance of their successful employment. As an important social network and capital resource, career social support may be highly influential in determining the success of college students’ employment.

Career social support is considered to be a significant and successful way into the labor market, and it also affects the resources available to individuals when considering, choosing and pursuing career choices (Chronister and Mcwhirter, 2004). Different to general social support, career social support is a domain-specific social support related to career-relevant tasks or issues. More specifically, it includes information and advice about career planning, financial support for job search behavior, comfort and encouragement after unsuccessful interviews, and other resources that individuals can obtain from their social networks such as parents, siblings, teachers, friends, and relatives (Hou et al., 2010; Jiang et al., 2017). Numerous empirical findings have shown that social support has significant positive impacts on college students’ employability (Michailidis et al., 2017), career self-efficacy (Wang and Fu, 2015), career maturity (Cho and Choi, 2007), and career preparation (Hirschi et al., 2011).

According to Resource Conservation Theory, people with more resources are less likely to suffer from resource loss attacks. Instead, they are more likely to gain resources (Hobfoll, 2001). Career social support and employability are both important personal resources and, according to the Career Construction Theory (CCT), career adaptability is a social psychological resource to cope with working tasks (Savickas, 1997). Individuals with more career social support are likely to improve their career adaptability, and then career adaptability and proactivity, which are regarded as a self-regulating resource together, influence the individual’s employability. Therefore, this study comprehensively investigates how these factors affect the employability of college students in the Chinese context.

### The Mediating Role of Career Adaptability

The term “Career adaptability” is considered a fundamental psychological resource enabling people to deal with present and anticipated tasks, transitions, and traumas in their occupational roles that change their social integration (Savickas, 1997), while concern, control, curiosity, and confidence constitute career adaptability resources (Savickas, 2005). Concern for the future enables individuals to think ahead and prepare for what might happen. Control enables a person to be more responsible for shaping themselves as well as the surroundings to meet future needs by adopting self-discipline and persistence. Curiosity facilitates a person to recognize themselves in different situations and roles. Confidence prompts the person to implement their life design (Savickas and Porfeli, 2012).

Considering the rapid change of the global market, it is important that people gain the competence to adapt to the requirements of career development and employment (Hou et al., 2012b). Career Construction Theory (Savickas, 2002) suggests that social support is an important contextual determinant of career adaptability. Previous research demonstrated a strong and consistent association between social support and career adaptability (Wang and Fu, 2015; Fawehinmi, 2016; Ghosh and Fouad, 2017). For example, in a sample of graduating seniors, Ghosh and Fouad (2017) indicated that social support can significantly predict the concern resource. In line with the statement of Ghosh and Fouad (2017), Wang and Fu (2015) recruited 879 Chinese college graduates as the sample and documented that career adaptability can be enhanced by social support.

According to Career Construction Theory, the adaption results are produced by adaptive readiness, adaptability resources, and adapting responses (Savickas and Porfeli, 2012). People who are willing (adaptive) and able (adaptability) to adjust behaviors according to changing conditions (adapting) were expected to have higher levels of adaptation (outcome). As a core component in career development, previous research suggested that career adaptability was a significant predictor for job satisfaction (Han and Rojewski, 2015), turnover intention (Chan and Mai, 2015), job performance (Ohme and Zacher, 2015), and employability (Mcardle et al., 2007; Dumulescu et al., 2015; Veld et al., 2016). Consequently, the first hypothesis is proposed:

Hypothesis 1: Career adaptability will play a mediating role between career social support and employability.

### The Moderating Role of Proactive Personality

Recent research has called attention to the joint contribution of social psychological capital and individual attributes to employability (Van der Heijden and Spurk, 2019). According to Resource Conservation Theory, a proactive personality can not only alleviate the adverse effect of resource scarcity, but also gain favorable effects when resources are sufficient. Accordingly, we explored whether the mediating role of career adaptability was moderated by a proactive personality. Proactive personality is deemed as a tendency to take personal initiative in a wide spectrum of activities and situations (Crant, 2000). High initiative individuals tend to create conditions for themselves, take the initiative to obtain relevant professional support, formulate their career planning, and pursue career goals they have set (Bateman and Crant, 1993). Previous studies have shown that these proactive career behaviors are beneficial to enhance individuals’ employability (De Vos and Soens, 2008; Chughtai, 2019).

A proactive personality may reinforce the effect of career adaptability on employability. On the one hand, a proactive personality facilitates promotion of career adaptability and personal growth. Individuals with proactivity are likely to be future-oriented, pay more attention to their career concerns, and obtain high self-efficacy in the world of work (Seibert et al., 1999; Tolentino et al., 2014). On the other hand, during difficult situations, proactive individuals often employ goal management strategies rather than risk management strategies. For instance, proactive individuals will choose a constructive way for further employability enhancement, and they often seek proactive information about occupations and their requirements (Brown et al., 2006), and they tend to take concrete measures to search for employment, commit to skill development, and thus get a job (Mcardle et al., 2007). In addition, for the employees with a proactive personality, they are more likely to develop and maintain high-quality relationships with their superiors and strengthen their employability (Li et al., 2010). Consequently, the second hypothesis is proposed as follows:

Hypothesis 2: A proactive personality will play a moderating role in the mediation effect of career adaptability between career social support and employability. Specifically, a proactive personality will moderate the second half path of the mediating link (i.e., the relationship between career adaptability and employability).

**Figure 1:**
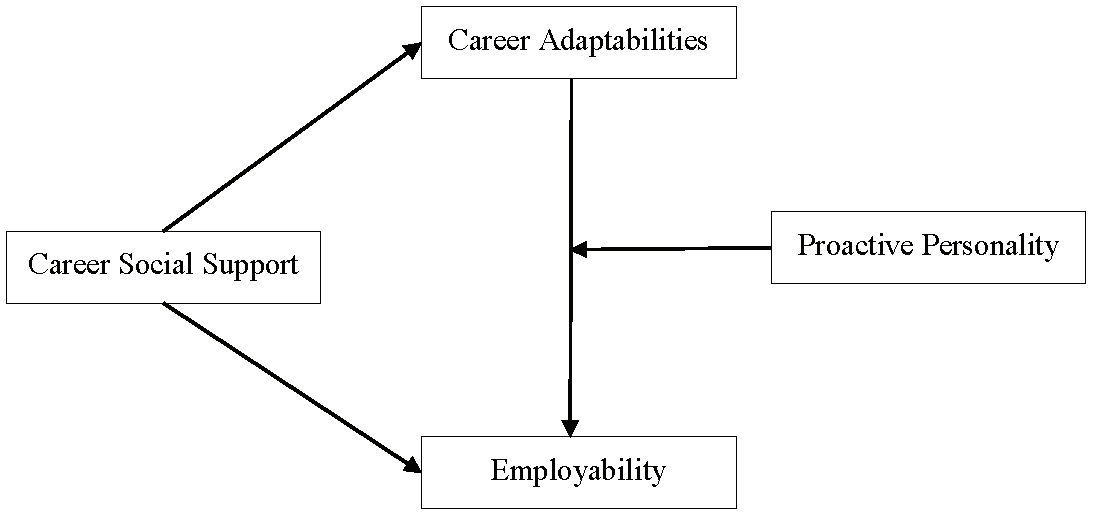
The conceptual model of the proposed moderated mediation framework.

In brief, we established a moderated mediation model (see Figure 1), and the purpose of this paper was twofold: (1) to explore whether career adaptability mediated the relationship between career social support and employability; (2) to test whether a proactive personality moderated the mediation effect of career adaptability. The moderated mediation model not only replies to the question of how career social support affects employability, but also provides a response when the enhancing effect is stronger or weaker.

## Methods

### Participants

Participants were 392 college students (*M*_age_ = 21.12 years, SD_age_ = 2.13, range = 19–24) recruited from two universities in China. Among the sample, 50.5% of these participants were sophomores, 40.6% were juniors, and 8.9% were seniors. Furthermore, 78.6% of these participants were female, 75.3% had one or more siblings, and 65.3% reported their place of residence as urban.

### Measures

*Career social support* was measured using the Career Social Support Inventory (CSSI) for Chinese College Students designed by Hou et al. (2010). The CSSI is composed of five subscales, namely parents’ support, siblings’ support, teachers’ support, friends’ support, and relatives’ support. Each subscale consists of 20 items measuring the following four types of support: information support (seven items), advice support (six items), emotional support (four items), and material support (three items). Sample items include, “tell me some information about college life in advance” (*information support*), “provide me with some advice about future employment and development” (*advice support*), “care about how I feel” (*emotional support*), and “provide me with tuition” (*material support*). Items were rated on a 5-point Likert scale ranging from 1 (*never*) to 5 (*very much*). The psychometric characteristics of the CSSI have been assessed in previous studies (e.g., Hou et al., 2012a). In this study, Cronbach’ *α* was 0.96.

*Career adaptability* was measured by means of the Career Adapt-Abilities Scale (CAAS; Savickas and Porfeli, 2012), which assessed four dimensions including concern (e.g., “Preparing for the future”), control (e.g., “Taking responsibility for my actions”), curiosity (e.g., “Becoming curious about new opportunities”), and confidence (e.g., “Working up to my ability”). Each subscale consisted of six items, and all items were rated on a 5-point Likert scale (1 = “*not strong*”, 5 = “*strongest*”). The CAAS has been previously validated in Chinese samples (e.g., Hou et al., 2012b; Hui et al., 2018). In the present study, Cronbach’ *α* was 0.91.

*Employability* was assessed using the University Student’s Employability Questionnaire (USEQ; Yu et al., 2014). This measure consisted of 36 items assessing eight dimensions covering interpersonal relationships (five items; Sample item: “I like to get along with others”), team cooperation (three items; Sample item: “I am willing to share information resources with team members”), learning ability (four items; Sample item: “I always want to learn new knowledge”), resolving problems (five items; Sample item: “I am able to properly handle multiple complex tasks”), social support (four items; Sample item: “My relatives can provide resources for my job hunting and career development”), network difference (three items; Sample item: “The people I interact with have very different hobbies and specialties”), an optimistic and open personality (six items; Sample item: “I will not be discouraged by failure”), and career identification (six items; Sample item: “I have clear employment goals”). All items were rated on a 5-point Likert scale ranging from 1 = *strongly disagree* to 5 = *strongly agree*. The USEQ has shown good reliability and validity in previous research (e.g., Ye et al., 2017). In this study, Cronbach’ *α* was 0.90.

*Proactive personality* was assessed by the means of the Proactive Personality scale (Bateman and Crant, 1993), which consisted of 10 items. Respondents were asked to rate each statement on a 5-point Likert scale ranging from 1 = *strongly disagree* to 5 = *strongly agree*. A sample item is “I often find new ways to improve my life”. The psychometric characteristics of this scale have been previously validated in Chinese samples (e.g., Kim et al., 2009). Cronbach’ *α* was 0.83 in the present study.

### Data Analysis

First, descriptive statistics and correlations were analyzed using SPSS version 24.0. Second, we used the PROCESS macro (Model 4; Hayes, 2013) of SPSS software to examine the indirect effect of career adaptability in linking career social support and employability. The bias-corrected bootstrapping method based on 2000 samples was used to test the significance of the indirect effect.

Third, we conducted a moderated mediation analysis using PROCESS (Model 14) to determine whether the indirect path was moderated by a proactive personality. Finally, we calculated conditional indirect effects so as to further test whether the indirect effect varied under the condition of different values of the moderating variable.

## Results

### Descriptive Statistics

Table 1 shows means, standard deviations, and correlations for all study indicators. As expected, career social support, career adaptability, and employability were positively correlated with each other, and proactive personality showed a positive relation to employability. Moreover, age significantly correlated with proactive personality, career adaptability, and employability. To avoid possible spurious effects, age was included as a control variable in the models.

**Table 1:** Means, standard deviations, and correlations of the variables.

	1	2	3	4	5	6	7	8
1. Age	—							
2. Gender	−0.06	—						
3. Only child	0.04	0.25^***^	—					
4. POR	0.12^***^	−0.10^*^	−0.46^***^	—				
5. CSS	0.06	0.08	0.08	−0.08	—			
6. CA	0.28^***^	0.09	0.08	−0.06	0.17^**^	—		
7. PP	0.25^***^	0.10	0.04	−0.12	0.15^**^	0.59^***^	—	
8. EM	0.31^***^	0.03	−0.01	−0.00	0.29^***^	0.65^***^	0.63^***^	—
*M*	21.12	0.79	0.75	0.35	2.53	3.60	3.48	3.33
SD	2.13	0.41	0.43	0.48	0.52	0.53	0.54	0.42

*n = 392. POR, place of residence; CSS, career social support; CA, career adaptability; PP, proactive personality; EM, employability. Gender was coded as 0 = male and 1 = female; Only child status was coded as 0 = no and 1 = yes; Place of residence was coded as 0 = rural and 1 = urban*.

* *p < 0.05*;

** *p < 0.01*;

*** *p < 0.001*.

### Testing for Mediation Effect

**Figure 2:**
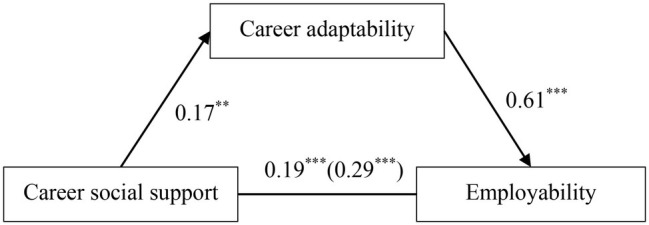
The mediating effect of career adaptability in the association between career social support and employability. ^**^p < 0.01, ^***^p < 0.001.

Next, we tested whether career adaptability mediated the relationship between career social support and employability. As seen in Figure 2, career social support significantly predicted employability [*b* = 0.29, SE = 0.05, 95%CI = (0.20, 0.39)] and career adaptability [*b* = 0.17, SE = 0.05, 95%CI = (0.07, 0.27)]. Furthermore, when career social support [*b* = 0.19, SE = 0.04, 95%CI = (0.11, 0.27)] and career adaptability [*b* = 0.61, SE = 0.04, 95%CI = (0.54, 0.69)] were entered as predictors, they both showed significant effects on employability. The bias-corrected percentile bootstrap method revealed significant indirect effect [indirect effect = 0.10, SE = 0.03, 95%CI = (0.04, 0.16)] and direct effect [direct effect = 0.19, SE = 0.04, 95%CI = (0.11, 0.27)]. The mediation effect accounted for 36.0% of the total effect. Thus, career adaptability played a partially mediating role in the relationship of career social support with employability, and both Hypotheses 1 and 2 were verified.

### Testing for Moderated Mediation Effect

**Figure 3:**
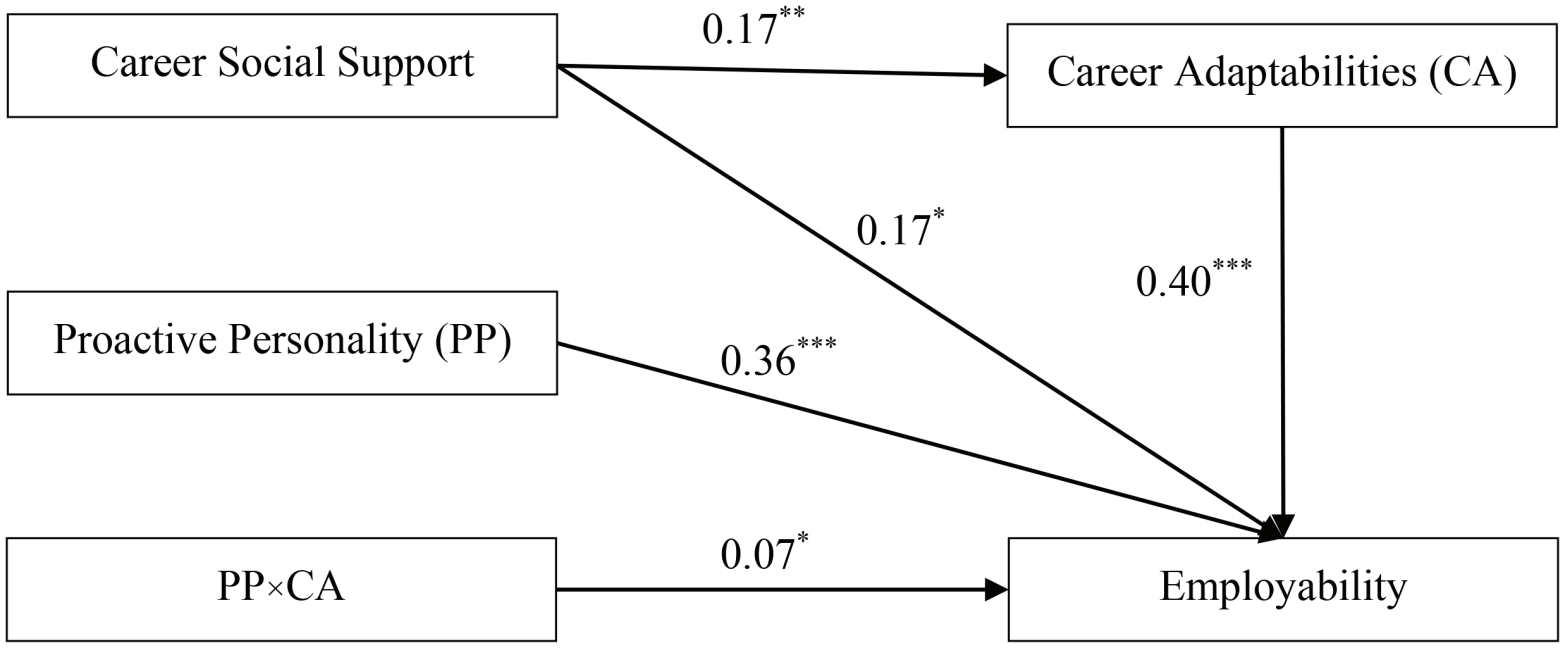
The moderating effect of proactive personality on the second stage of the indirect association. ^*^p < 0.05, ^**^p < 0.01, ^***^p < 0.001.

We examined whether a proactive personality moderated the mediating effect of career adaptability. Results from Figure 3 indicated that (1) career social support significantly predicted career adaptability [*b* = 0.17, SE = 0.05, 95%CI = (0.07, 0.27)], and (2) both career adaptability [*b* = 0.40, SE = 0.04, 95%CI = (0.32, 0.49)] and the interaction effect of career adaptability and a proactive personality [*b* = 0.07, SE = 0.03, 95%CI = (0.01, 0.13)] significantly affected employability. The findings suggested that the indirect effect between career social support and employability was moderated by a proactive personality. More specifically, a proactive personality moderated the second half path of the mediating effect. Thus, Hypotheses 3 was supported.

For clearly demonstrating the moderating role of proactive personality, this study conducted simple slope tests. The results revealed that higher career adaptability was strongly related to higher employability (*b*_simple_ = 0.47, *t* = 10.42, *p* < 0.001) under the condition of a high proactive personality (i.e., one SD above the mean). The relation between adaptability and employability was much weaker (*b*_simple_ = 0.33, *t* = 7.47, *p* < 0.001), however, in the condition of a low proactive personality (i.e., one SD below the mean).

Finally, the test of conditional indirect effects showed that the total indirect effect was more noteworthy for college students with high levels of a proactive personality [indirect effect = 0.08, SE = 0.03, 95%CI = (0.03, 0.14)], than for those with low levels of proactive personality [indirect effect = 0.06, SE = 0.02, 95%CI = (0.02, 0.10)]. Furthermore, the index of moderated mediation (Hayes, 2015) was 0.011 [SE = 0.008, 95%CI = (0.001, 0.030)], indicating that proactive personality strengthened the mediating effect of career adaptability.

## Discussion

With a steady increase in the number of university graduates, the employment of college students has become a common concern of higher education and even society as a whole in China. This study extended previous research by establishing a moderated mediation model, which systematically tested the synergistic impacts of individuals (i.e., career adaptability and proactive personality) and contextual (i.e., career social support) factors on college students’ employability.

First, in accordance with Resource Conservation Theory (Hobfoll et al., 2003) and previous empirical findings (European Training Foundation, 2002; Fabio and Kenny, 2015), the current study verified the positive function of career social support in cultivating college students’ employability. The Resource Conservation Theory emphasizes the links between individuals’ conditional resources (e.g., social support obtained from important others) and the chance of their successful employment (i.e., employability). Meanwhile, our results verify the view that the more powerful an individual’s social support network, the stronger his or her employability (Michailidis et al., 2017).

Second, this study identified that career adaptability mediated the association between career social support and employability, indicating that establishing a good social support system is conducive to the cultivation of career adaptability of college students (Creed et al., 2009), which in turn improves their employability (Koen et al., 2012; Kang et al., 2016). Thus, career adaptability could serve as a “bridge” linking career social support and employability, and career adaptability should be considered as a valuable psychosocial asset which may be fostered by providing diverse aspects of social support (e.g., information support, advice support, emotional support, and financial support).

Third, this study indicated that college students with high levels of a proactive personality were more likely to approach powerful employability. More importantly, we found that a proactive personality strengthened the salutary effect of career adaptability on employability. This implies that college students with higher proactivity may still take advantage of all the opportunities to actively seek employment information related to their career development, take the initiative to seize practical training, and further improve their employability, when they have stable career adaptability. Conversely, for those with lower proactivity, even if they have enough confidence in facing various challenges, they could not find and make use of beneficial opportunities in a timely manner. Thus, it is impossible to further identify what professional skills they need to improve, which in turn reduces the increase in employability (Fawehinmi, 2016).

The moderating role of proactive personality may be explained in two ways. First, Social Cognitive Theory suggests the individuals’ behavior depends on their self-efficacy, and high levels of proactivity is likely to enhance individuals’ career confidence and make them spend more energy on career exploration, and thus improve their employability (Cai et al., 2015). Second, Self-regulation Theory believes that individuals can adjust their views, emotions, and behaviors based on the outcomes of their action, and that individuals with high levels of proactivity are more likely to adjust their career concerns and control their career behavior based on the outcomes, and thus contribute to career success (Merino-Tejedor et al., 2016). These findings indicate that college students should improve their career adaptability and proactivity simultaneously so as to gain powerful employability.

Despite the importance of the findings, some limitations deserve to be mentioned. First of all, the study relied on a convenience sample of Chinese college students, and the mean age of the sample was only 21.12 years, which limited the generalizability of results. As such, further research should be designed to replicate the current study to samples consisting of other cultures and age groups. Second, the data we collected was at a single point in time. Long-term and continuous studies should be conducted to examine the causal links among career social support, career adaptability, and employability. Finally, the current study used college students self-report to collect data, and further studies would benefit from assessing the constructs using multiple informants.

Despite the above-mentioned limitations, this research has several practical implications. To begin with, the present study corroborates the importance of career social support. Supports from family, teachers, and friends may help college students to gain more valuable employment information and financial assistance, which is beneficial for college students’ successful employment. Therefore, career social support should become a focus for future employability intervention plans. Besides the external support, college students should grow awareness of developing their own career adaptability persistently. On the other hand, this study offers preliminary evidence that a proactive personality enhances the significant influence of career adaptability on employability. Accordingly, we should not overlook the protective roles of personality trait resources (e.g., proactive personality).

In conclusion, this research deepens the understanding of how and when good career social support increases college students’ employability through considering individual (career adaptability and proactive personality) and contextual (career social support) variables simultaneously. Overall, good career social support can promote college students’ career adaptability, which in turn facilitates employability. Moreover, the beneficial role of career adaptability is strengthened by high levels of a proactive personality.

## Data Availability Statement

The datasets generated for this study are available on request to the corresponding author.

## Ethics Statement

The study protocol was approved by the Hunan Normal University Research Ethnics Committee. All participants gave written informed consent.

## Author Contributions

HG and YH designed the study. HG, QZ, and YC collected data, developed, and performed the statistical analysis in conjunction. TX and HG wrote the first draft of the manuscript and revised the manuscript.

### Conflict of Interest

The authors declare that the research was conducted in the absence of any commercial or financial relationships that could be construed as a potential conflict of interest.
